# Exploring neutrophil extracellular traps: mechanisms of immune regulation and future therapeutic potential

**DOI:** 10.1186/s40164-025-00670-3

**Published:** 2025-05-29

**Authors:** Fan Gao, Hongwei Peng, Ruixue Gou, Yulan Zhou, Simei Ren, Fei Li

**Affiliations:** 1 Jiangxi Provincial Key Laboratory of Hematological Diseases, Department of Hematology, The First Affiliated Hospital, Jiangxi Medical College, Nanchang University, Nanchang, Jiangxi China; 2 Jiangxi Clinical Research Center for Hematologic Disease, Nanchang, China; 3 Institute of Lymphoma and Myeloma, Nanchang University, Nanchang, China; 4 Department of Pharmacy, The First Affiliated Hospital of Nanchang University, Nanchang, Jiangxi China; 5 National Center for Clinical Laboratories, Beijing Engineering Research Center of Laboratory Medicine, Beijing Hospital, National Center of Gerontology, Institute of Geriatric Medicine, Chinese Academy of Medical Sciences, Chinese Academy of Medical Sciences and Peking Union Medical College, Beijing, People’s Republic of China

**Keywords:** Neutrophil extracellular traps, Immune cells, Immune response, Therapeutic strategy

## Abstract

Neutrophil extracellular traps (NETs) are complex, web-like structures consisting of DNA intertwined with antimicrobial proteins, which neutrophils release upon immune activation. These structures play a crucial role in pathogen elimination, particularly in infectious diseases. However, their involvement in various pathological conditions is multifaceted and context-dependent, while NETs contribute to host defense against infections, they can also exacerbate sterile inflammation, autoimmune disorders, and tumor progression. This review provides a comprehensive analysis of the molecular mechanisms governing NET formation and examines their interactions with immune cells, emphasizing how these interactions shape immune responses and drive disease dynamics. Furthermore, it explores ongoing clinical trials and emerging therapeutic strategies targeting NETs, offering critical insights into their potential translational applications in clinical practice.

## Introduction

Neutrophils constitute 50%–79% of circulating leukocytes and serve as the frontline defenders against invading pathogens [1]. Recent investigations have revealed a remarkable heterogeneity among them, characterized by a wide spectrum of phenotypic and functional attributes [2, 3]. In the context of malignant tumors, elevated neutrophil counts have been linked to poor clinical outcomes [4]. In 2009, tumor-associated neutrophils (TANs) were first categorized into two distinct phenotypes, N1 (anti-tumor) and N2 (tumor-promoting), based on their functional roles within the tumor microenvironment (TME) [5–7].

Both N1 and N2 neutrophils have been shown to generate neutrophil extracellular traps (NETs) [8]. The role of NETs in malignancies remains a subject of ongoing debate, largely due to their context-dependent effects shaped by the TME and cancer type [9, 10]. Since their initial discovery in 2004 as antimicrobial structures, research has predominantly focused on their pathogen-trapping capabilities, with numerous microorganisms identified as primary inducers of NET formation [11]. However, it has become increasingly clear that uncontrolled NET generation, often triggered by heightened immune responses or chronic inflammation, can lead to significant tissue injury [12]. Recent studies have further highlighted the dualistic nature of NETs in non-infectious diseases: on the one hand, NETs serve as autoantigens that exacerbate autoimmune disorders by fueling tissue destruction and sustained inflammation [13]; on the other, they can mitigate sterile inflammation by degrading pro-inflammatory mediators, thereby contributing to the resolution of inflammation and facilitating tissue repair [14]. While the biological functions of NETs are well-documented, their intricate interactions with the immune microenvironment, including cytokines, complement components, and immune cells, suggest they play a pivotal regulatory role in both innate and adaptive immunity. This review aims to provide a comprehensive analysis of NET-immune microenvironment interactions and explore emerging therapeutic strategies targeting NETs in disease management.

## Mechanisms of NET formation and regulation

### Stimuli inducing NET formation: pathogens and other triggers

Initially regarded solely as a defense mechanism against bacterial infections, the functional landscape of NETs has significantly broadened with advancing research. Beyond bacteria, a wide array of pathogens, including fungi, viruses, and parasites, have been identified as potent NET inducers. Moreover, NET formation is now increasingly implicated in sterile inflammatory conditions, autoimmune disorders, and malignancies. As summarized in Table 1, while NETs play a crucial protective role in combating microbial infections, their excessive or dysregulated production can exacerbate pathological conditions, as exemplified by their contribution to the severity of diseases such as COVID-19. Although most known NET inducers are exogenous, the role of endogenous factors within neutrophils that initiate NET formation remains poorly understood. Consequently, the precise molecular mechanisms governing NET induction in various pathological contexts require further elucidation.

**Table 1:** Stimulators of extracellular trap formation in neutrophils

Type	Stimulus	Benefit/Harm	References
Pathogens	Streptococcus suis	B	de Buhr et al. [15]
	S. aureus	B	Focken et al. [16]
	C. albicans	B	Guiducci et al. [17]
	Group B Streptococcus	B	Boldenow et al. [18]
	P. aeruginosa	B	Thanabalasuriar et al. [19]
	L. amazonensis	B	DeSouza-Vieira et al. [20]
	T. gondii	B	Villagra-Blanco et al. [21]
	Escherichia coli	B	Lin et al. [22]
	Salmonella typhimurium	B	Babatunde et al. [23]
	Francisella tularensis	B	Pulavendran et al. [24]
	Yersinia pseudotuberculosis	B	Casutt-Meyer et al. [25]
	Hantavirus	B	Raftery et al. [26]
	Dengue virus	H	Sung et al. [27]
	Chikungunya virus	B	Hiroki et al. [28]
	Respiratory syncytial virus	B	Muraro et al. [29]
	Hepatitis B virus	H	Zhan et al. [30]
	SARS-CoV-2	H	Veras et al. [31]
	Influenza virus	B	Koupenova et al. [32]
	Sendai virus	H	Akk et al. [33]
Immunological stimuli	IL-1β	H	Keshari et al. [34]
	TNFα	H	Keshari et al. [34]
	IL-8	H	Nie et al. [35]
	IL-17	H	Zhang et al. [36]
	IL-33	H	Jin et al. [37]
	Midkine	H	Weckbach et al. [38]
	LPS	H	Tong et al. [39]
	HMGB1	H	Maugeri et al. [40]
	P-selectin	H	Etulain et al. [41]
	Ang1 and Ang2	H	Lavoie et al. [42]
	fMLP	H	Marino et al. [43]
	Monosodium urate	H	Desai et al. [44]
	Calcium carbonate crystals	H	Leppkes et al. [45]
	Amyloid-β	H	Zenaro et al. [46]
	Immune complexes	H	Kessenbrock et al. [47]
Tumor-associated stimuli	CXCR1 and CXCR2	H	Teijeira et al. [48]
	G-CSF	H	Demers et al. [49]
	C3a	H	Guglietta et al. [50]
Chemical or physical factors	PMA	-	Douda et al. [51]
	Cigarette smoke	H	Qiu et al. [52]
	PM2.5	H	He et al. [53]
	Bleomycin	H	Suzuki et al. [54]
	Nanoparticles	B	Bilyy et al. [55]
	A23187(Calcium ionophore)	B	Kenny et al. [56]
	Acrolein	B	Luo et al. [57]
Other	Endothelial cell	H	Zhang et al. [58]
	Platelet	H	McDonald et al. [59]
	Gut microbiome dysbiosis	H	Tian et al. [60]
	oxLDL	H	Obama et al. [61]

### Canonical regulatory pathways: core mechanisms of NET formation

**Fig. 1:**
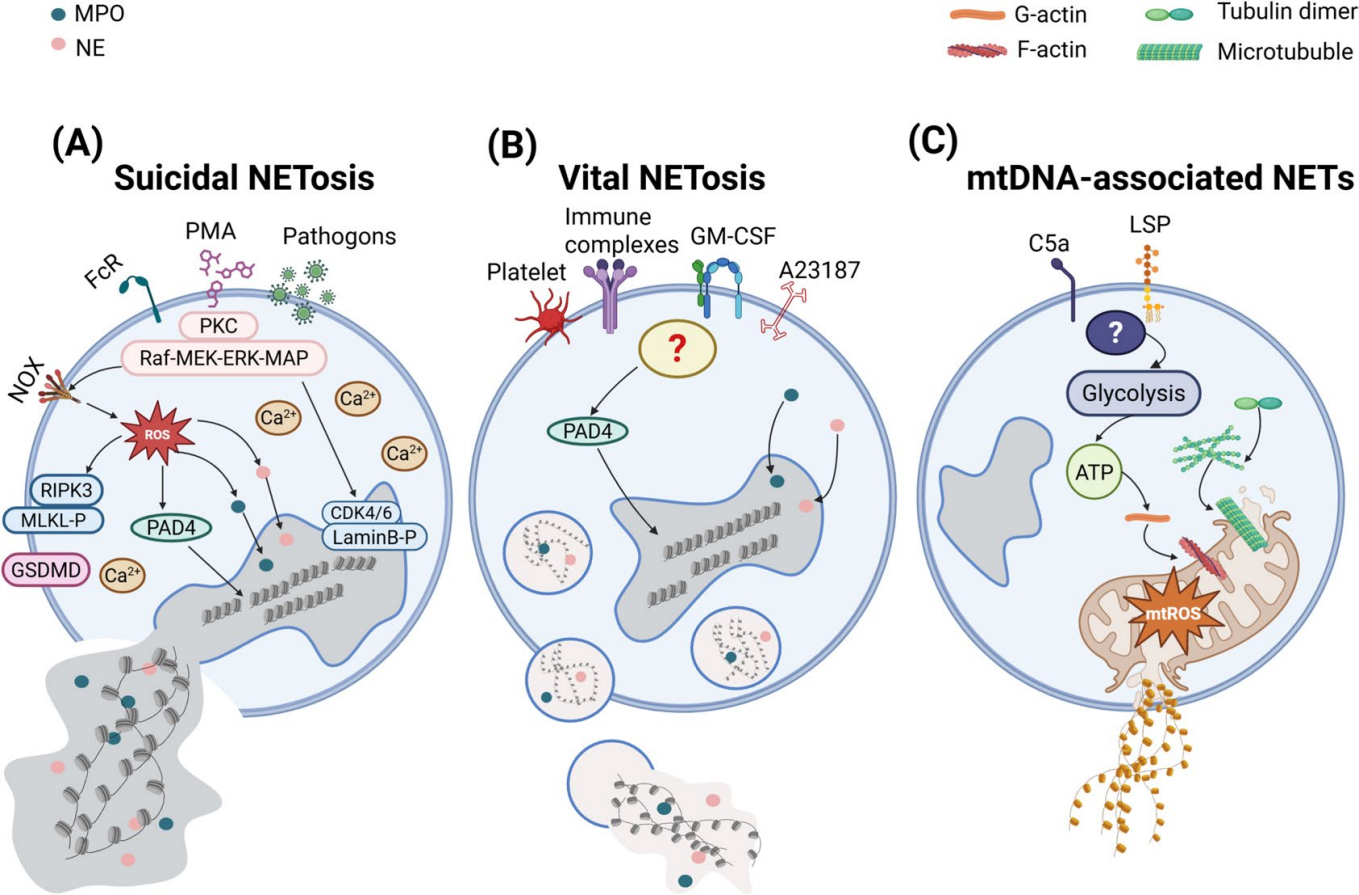
Mechanisms underlying NET formation. The genesis of NETs can be delineated into three principal mechanisms. The initial pathway, termed "suicidal NETosis", commences with nuclear membrane disintegration, followed by the dissolution of cellular polarity, chromatin decondensation, and ultimately, cell membrane rupture leading to cell death. A second pathway, identified as "vital NETosis", involves the encapsulation of decondensed chromatin within vesicles that are extruded from the cell, a process that does not entail cell demise. The final mechanism is mitochondrial DNA-dependent NETosis, which, akin to viable NETosis, is not associated with cell death and is specific to the viable NET formation

NET formation is governed by three major regulatory pathways, each controlled by distinct molecular mechanisms and contextual signals (Fig. 1). Below, we detail the core regulatory processes orchestrating these pathways and their intricate interconnections within the broader NET signaling network.

#### NOX-Dependent NETosis: ROS-Mediated Regulation

The first and best-characterized pathway of NET formation is the NADPH oxidase (NOX)-dependent mechanism, commonly referred to as "suicidal NETosis" (Fig. 1A). This process is triggered by stimuli such as phorbol 12-myristate 13-acetate (PMA) [51] and microbial pathogens [62], which activate protein kinase C (PKC) and the Raf-MEK-ERK-MAPK signaling cascade [63]. This activation leads to an increase in cytoplasmic calcium levels, facilitating the assembly of the NOX complex, which rapidly generates ROS [64, 65].

Reactive oxygen species (ROS) initiate the activation of neutrophil elastase (NE), which translocates into the nucleus to degrade histones and facilitate chromatin decondensation [66]. This process is further amplified by myeloperoxidase (MPO), ultimately culminating in plasma membrane rupture and the extracellular release of NETs [67]. Concurrently, peptidylarginine deiminase 4 (PAD4) catalyzes the citrullination of nuclear histones, thereby promoting additional chromatin relaxation. Following the breakdown of the nuclear envelope, citrullinated histones and DNA are expelled into the cytoplasm, where they are modified by granule-derived proteins and cytoplasmic enzymes to form mature NETs [68–70].

Nuclear envelope rupture in this pathway is largely mediated by cyclin-dependent kinases 4 and 6 (CDK4/6), which phosphorylate retinoblastoma protein (Rb) and lamin B, thereby destabilizing nuclear integrity [71]. Furthermore, elevated ROS levels activate receptor-interacting serine/threonine-protein kinase 3 (RIPK3) and mixed lineage kinase domain-like (MLKL) protein, both of which contribute to plasma membrane rupture [44]. In the final stages of NET release, gasdermin D (GSDMD) plays a crucial role in increasing membrane permeability, facilitating cell lysis and the extracellular expulsion of NETs [72].

#### NOX-independent NETosis: chromatin remodeling via PAD4 activation

The second pathway of NET formation operates independently of NOX and ROS, instead relying on the activation of PAD4. This process, termed "vital NETosis", allows neutrophils to remain functional post-NET release. Various stimuli, including granulocyte–macrophage colony-stimulating factor (GM-CSF) [73], activated platelets [59], A23187 [56], and immune complexes [47, 74], can rapidly trigger this pathway (Fig. 1B). In this mechanism, chromatin undergoes decondensation, becomes decorated with granule proteins and histones, and is subsequently encapsulated within vesicles that bud off from the nucleus. These vesicles are then expelled, forming extracellular NETs while preserving neutrophil viability. This unique mode of NET release underscores a critical distinction from NOX-dependent pathways, as it enables neutrophils to continue participating in immune surveillance and defense.

#### Mitochondrial DNA-driven NETosis: metabolic priming and inflammatory amplification

A third, distinct mechanism involves NETs enriched with mitochondrial DNA rather than nuclear DNA (Fig. 1C) [73, 75]. Specific stimuli, such as complement component 5a (C5a) and lipopolysaccharide (LPS), can trigger neutrophils to rapidly release mitochondrial DNA, a process that is heavily dependent on mitochondrial ROS generation [73, 76]. Unlike NOX-mediated NETosis, this pathway does not require neutrophil lysis but instead depends on glycolytic adenosine triphosphate (ATP) production, which orchestrates cytoskeletal reorganization via microtubule and F-actin remodeling, both essential for mitochondrial DNA release and granule degranulation [77].

Beyond its structural role in NETs, mitochondrial DNA is also implicated in inflammatory signaling. It serves as a ligand for the cyclic GMP-AMP synthase/stimulator of interferon genes (cGAS/STING) pathway, which activates innate immune responses and has been linked to the pathogenesis of autoimmune diseases such as systemic lupus erythematosus (SLE) [78]. These findings highlight the multifaceted role of mitochondrial DNA in NET formation and its contribution to inflammation-driven pathologies.

### The role of autophagy in NET regulation: synergistic mechanisms

**Fig. 2:**
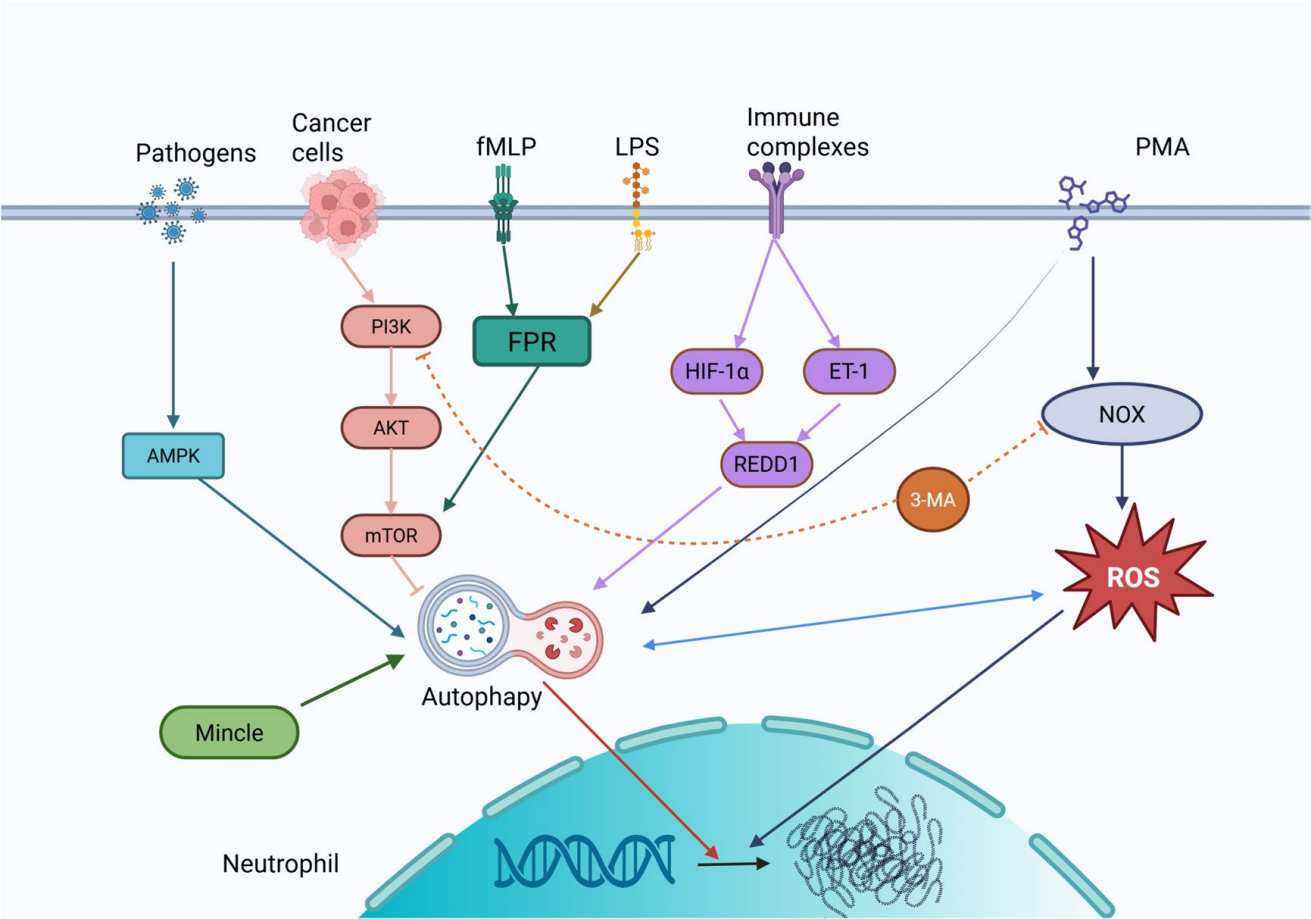
Regulation of NET formation by autophagy signaling pathway. Autophagy within cells synergistically influences the formation of NETs, with the process of NET formation being contingent upon cellular autophagy

Beyond the classical NETosis pathways, autophagy has emerged as a pivotal modulator of NET formation through intricate crosstalk with key signaling networks. Itakura et al. [79] have demonstrated that autophagy facilitates NET release, whereas its inhibition markedly suppresses NET formation (Fig. 2). The mechanistic target of rapamycin (mTOR) pathway serves as a central link between autophagy and NET regulation: activation of autophagy by rapamycin enhances NET production, whereas mTOR activation, such as that observed in melanoma, suppresses it [80]. Within this framework, the PI3K/mTOR axis operates as a critical regulatory hub, with distinct PI3K isoforms exerting differential effects on NET formation [81].

Pharmacological investigations further underscore the context-dependent nature of autophagy-mediated NET regulation. In models of ANCA-associated vasculitis and leukemia, treatment with 3-methyladenine (3-MA) inhibits PI3K class III-mediated autophagy, leading to a reduction in NET formation [82]. However, 3-MA fails to suppress rapamycin-induced NET release, suggesting a bifurcated regulatory mechanism wherein 3-MA primarily impedes ROS-dependent NETosis by inhibiting the PI3K class I/NADPH oxidase pathway [117].

Clinically, the interplay between autophagy and NET formation has been implicated in the pathogenesis of various inflammatory disorders. In SLE and ulcerative colitis, upregulation of the stress-response mediator REDD1 promotes both autophagy activation and NET release, thereby amplifying IL-1β-driven inflammatory cascades [83, 84]. This REDD1–autophagy–NET axis exemplifies how cellular stress pathways can perpetuate chronic inflammation and exacerbate disease progression.

The intricate regulatory network connecting autophagy and NETs highlights three key research priorities as follows: (1) temporal regulation of autophagic flux throughout NET formation; (2) cell-type-specific signaling adaptations in distinct pathological contexts; (3) therapeutic strategies that selectively target pathogenic NETs while preserving their physiological roles in host defense. Leveraging single-cell multi-omics and conditional knockout models may pave the way for precision medicine approaches to modulate NETs in inflammatory and autoimmune diseases while minimizing off-target effects.

## Crosstalk between NETs and adaptive immunity

### NETs induce CD8⁺ T-cell exhaustion

**Fig. 3:**
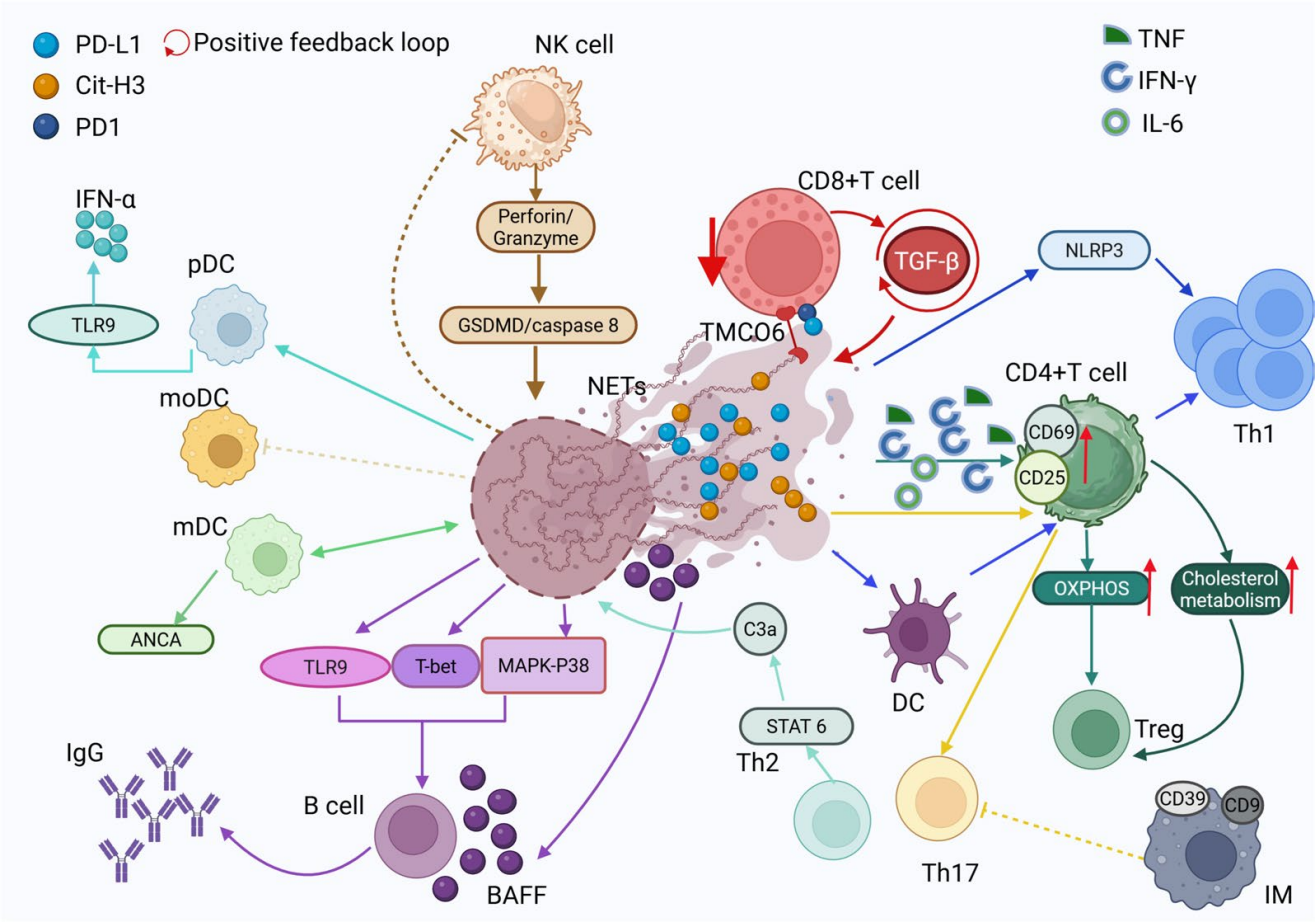
Schematic Representation of NET-Lymphocyte Crosstalk. NETs exert complex effects on the immune landscape, leading to the depletion and dysfunction of CD8^+^ T cells, influencing the differentiation of CD4^+^ T cells, and impairing the antitumor efficacy of NK cells. Additionally, DCs play a role in antigen presentation derived from NETs, further modulating immune responses

NETs contribute to CD8⁺ T-cell exhaustion through a multifaceted interplay of metabolic dysregulation, immune checkpoint activation, and physical barriers that restrict T-cell infiltration. Clinical studies in solid tumors, such as non-small cell lung cancer, and murine models of liver metastasis demonstrate a strong correlation between elevated circulating NETs, upregulation of IL-8, and CD8⁺ T-cell dysfunction [85] (Fig. 3). Additionally, these cells undergo metabolic reprogramming, characterized by diminished mitochondrial function, reduced glucose uptake, and an increased reliance on fatty acid metabolism, phenotypes that can be reversed through DNase-mediated NET degradation [86].

At the molecular level, NET-associated PD-L1 directly inhibits T-cell receptor signaling, while arginase 1 (Arg1) within NETs depletes extracellular arginine, further compromising T-cell proliferation and cytotoxic function [86, 87]. Additionally, NETs induced by TGF-β1 interact with transmembrane and coiled-coil domain-containing protein 6 (TMCO6) on CD8⁺ T cells via DNA binding, triggering apoptosis and amplifying TGF-β1 secretion in a self-perpetuating loop [88] (Fig. 3). Beyond their molecular immunosuppressive roles, NETs also form a dense extracellular matrix at the tumor-stroma interface, creating a physical barrier that impedes CD8⁺ T-cell infiltration [89].

Targeting NETs has emerged as a promising therapeutic strategy in cancer immunotherapy. Combination approaches using DNase or PAD4 inhibitors alongside immune checkpoint blockade (e.g., anti-PD-1 therapy) have shown potential in reversing CD8⁺ T-cell exhaustion and restoring antitumor immunity, underscoring the translational significance of NET-targeting interventions in oncology.

### NETs modulate naïve CD4⁺ T-cell differentiation

NETs profoundly influence the differentiation of naïve CD4⁺ T cells by modulating metabolic and transcriptional pathways, leading to disease-specific outcomes. In metabolic disorders such as nonalcoholic steatohepatitis (NASH) and sepsis, NETs promote oxidative phosphorylation (OXPHOS) and cholesterol metabolism in naïve CD4⁺ T cells, thereby driving the differentiation of FOXP3⁺ regulatory T cells (Tregs) through TLR4 signaling [90–93]. Conversely, in autoimmune diseases like rheumatoid arthritis (RA), NET-activated dendritic cells (DCs) facilitate T helper 1 (Th1) polarization, while NET-derived histones directly engage TLR2 on T cells, biasing differentiation toward the Th17 lineage, particularly in periodontitis [52, 94–97].

In the context of cancer, breast cancer lung metastases induce signal transducer and activator of transcription 6 (STAT6)-dependent Th2 cytokine production, which upregulates complement C3 in lung stromal cells. This, in turn, recruits neutrophils and amplifies NET formation, establishing a feedforward loop that exacerbates metastatic progression [96] (Fig. 3).

Interestingly, endogenous mechanisms exist to counteract NET-mediated immunomodulation. In neutrophilic asthma, CD39⁺CD9⁺ interstitial macrophages suppress NET formation, thereby inhibiting Th17 differentiation [97, 98].

These findings position NETs as critical orchestrators of CD4⁺ T-cell plasticity, dynamically shaping immune responses through metabolic and transcriptional reprogramming. By fine-tuning the balance between immune defense and pathological inflammation, NETs play a dual role in health and disease, offering potential therapeutic targets for autoimmune disorders, metabolic diseases, and cancer.

### NETs drive antigen-specific responses in B lymphocytes

NETs play a pivotal role in B-cell activation and autoantibody production in autoimmune diseases by facilitating antigen presentation, cytokine crosstalk, and neoantigen generation [158]. In SLE, NET-derived LL37–DNA complexes form immune complexes that activate TLR9 signaling in polyclonal B cells, leading to the expansion of autoreactive memory B cells targeting LL37. Additionally, NETs release B-cell activating factor (BAFF), promoting B-cell survival and enhancing humoral immune responses [99–101]. In lupus nephritis, B cells internalize NET-derived material, inducing T-bet transcriptional activation and driving the production of IgG2 antibodies [102]. Furthermore, citrullinated histones within NETs act as persistent antigenic stimuli, promoting the generation of anticitrullinated protein antibodies (ACPA) in synovial ectopic germinal centers, a process exacerbated by IL-8–mediated neutrophil recruitment and complement activation [103, 104].

This self-sustaining cycle of NET-driven B-cell activation and autoantibody generation underscores their central role in autoimmune pathogenesis. Emerging therapeutic strategies targeting NET/B-cell interactions, including TLR9 inhibition and BAFF blockade, hold promise for disrupting pathogenic autoimmune cascades in diseases such as SLE and RA.

## Crosstalk between NETs and innate immunity

### Reciprocal regulation between NETs and natural killer (NK) cells

NK cells are key effectors of cytotoxic immunity, eliminating infected or malignant cells through perforin/granzyme-mediated lysis while modulating immune responses via IFN-γ secretion. Their interaction with NETs establishes a bidirectional regulatory loop, which can be either protective or pathogenic, depending on the disease context [105].

In HBV-associated liver failure, NK cells induce hepatocyte pyroptosis through a GSDMD/caspase-8–dependent perforin/granzyme B pathway, leading to the release of HMGB1, which subsequently activates neutrophils and promotes NET formation. NK cell-derived cytokines, including IFN-γ, GM-CSF, and TNF-α, further prolong neutrophil survival and enhance NET production [106–111]. Although pyroptosis and NET formation are distinct processes, they are closely interconnected in this setting. Inflammatory factors released during pyroptosis can indirectly trigger NET release, while NETs exacerbate inflammation and facilitate the progression of pyroptosis, together contributing to the pathogenesis of HBV-associated liver failure. Notably, disulfiram, an aldehyde dehydrogenase inhibitor, exhibits anti-inflammatory and anticancer properties by simultaneously limiting both NET formation and pyroptosis [112].

Conversely, within the TME, NETs actively suppress NK-cell cytotoxicity through multiple mechanisms. In colon cancer, NETs promote src kinase-associated phosphoprotein 1 (SKAP1)-mediated NFATc1/CXCL8 signaling, which dampens NK-mediated tumor killing [113]. In esophageal carcinoma, CD276 overexpression induced by NETs depletes NK-cell populations, further weakening antitumor immunity [114] (Fig. 3). Additionally, NETs facilitate platelet-derived TGF-β release, which establishes an immunosuppressive niche that inhibits NK function [115, 116].

Notably, the NET-NK axis appears to be bidirectionally regulated in a context-dependent manner. Murine models with impaired NET formation exhibit reduced NK-cell numbers, suggesting that NETs may play a role in maintaining NK-cell homeostasis [111]. However, the precise spatiotemporal dynamics of this feedback loop remain poorly understood, whether NETs directly modulate NK recruitment, activation thresholds, or exhaustion phenotypes requires further investigation.

### DCs process NET-derived antigens

DCs, particularly plasmacytoid DCs (pDCs) and myeloid DCs (mDCs), serve as a crucial link between NET immunogenicity and adaptive immune responses [117]. In autoimmune disorders, NET-derived DNA complexes activate pDCs via TLR9, amplifying IFN-α-driven inflammation in SLE and other rheumatic diseases [118, 119]. In contrast, the severity of chronic obstructive pulmonary disease (COPD) correlates with NET-induced NF-κB cytokine cascades, a mechanism independent of IFN-α, highlighting the disease-specific nature of DC activation [120]. Mechanistically, mDCs form stable interactions with NETs, internalizing neutrophil-derived antigens and subsequently initiating the production of ANCA and anti-dsDNA autoantibodies. Importantly, this process depends on the preservation of intact NET architecture, highlighting the critical role of NET integrity in modulating immune responses [121].

Beyond their pathological roles, NET–DC interactions also offer therapeutic potential. For example, NETs generated in leukemia display mutant nucleophosmin 1 (NPMc⁺) antigens, which can be leveraged to prime DC-based cancer vaccines, providing a promising strategy to combat myeloid hyperplasia [122]. This dual role of NETs, as pathogenic drivers in autoimmunity and adjuvants in oncology, emphasizes the pivotal importance of antigenic context (Fig. 3). Future research must elucidate how NET composition (e.g., oxidized vs. non-oxidized DNA, proteolytic cargo) influences DC activation thresholds and T-cell polarization, thereby paving the way for precision-targeted immunotherapies and vaccine development.

### Macrophages and NETs engage in complex interactions

#### Macrophages mediate NET clearance

Macrophages, derived from bone marrow progenitors that circulate as monocytes before differentiating into tissue-resident macrophages, play key roles in pathogen clearance, antigen presentation, and tissue homeostasis [123]. Functionally, macrophages polarize into two distinct phenotypes: M1 (classically activated macrophages), which are primarily involved in pro-inflammatory responses and pathogen elimination; M2 (alternatively activated macrophages), which are associated with anti-inflammatory functions, tissue repair, and immune regulation [124].

**Fig. 4:**
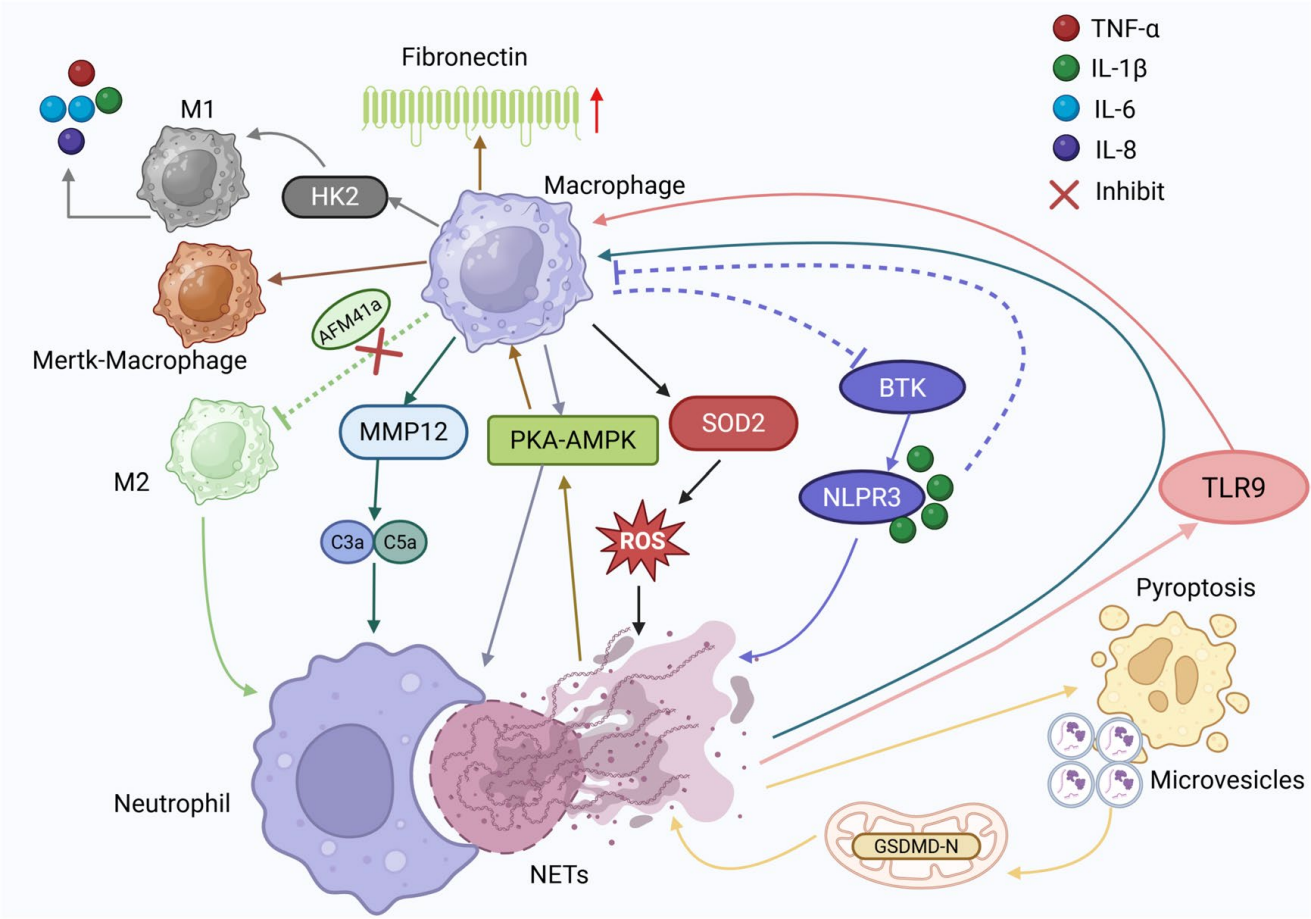
Schematic representation of the interplay between NETs and macrophages. Macrophages contribute to NET formation by secreting cytokines and are involved in the clearance of NETs through phagocytosis. In turn, NETs modulate macrophage polarization and function, highlighting the bidirectional relationship between these two cellular components

Pro-inflammatory M1 macrophages promote NET clearance through a process resembling macropinocytosis, priming themselves for NET engulfment and subsequent degradation via phagocytosis. Notably, in human abdominal aortic aneurysms, macrophage density inversely correlates with circulating NET levels, underscoring their critical role in maintaining NET homeostasis and facilitating inflammation resolution [125] (Fig. 4). A key mechanism underlying NET degradation involves matrix metalloproteinase-12 (MMP12), identified by Bellac et al. [126] as a major mediator of macrophage-driven NET clearance. MMP12 not only prevents excessive complement activation by degrading C3 but also neutralizes the anaphylatoxins C3a and C5a, thereby mitigating inflammation. Moreover, MMP12 enhances phagocytic efficiency by cleaving iC3b and C3b modulators, fine-tuning complement activity to avert uncontrolled immune activation.

Additionally, the AMP-activated protein kinase (AMPK) pathway enhances macrophage-mediated NET clearance, providing a potential therapeutic avenue for mitigating acute respiratory distress syndrome (ARDS) [127]. Following NET phagocytosis, macrophages degrade NET components via cytoplasmic nucleic acid exonucleases, including DNases, ensuring complete clearance and preventing persistent immune activation.

Future research should aim to elucidate the distinct roles of M1 and M2 macrophages in NET processing across diverse disease contexts, investigate how macrophage-mediated NET degradation influences the resolution of inflammation and the development of autoimmunity, and explore therapeutic strategies targeting molecules such as MMP12 and AMPK to enhance NET clearance and mitigate pathological inflammation.

#### Macrophages facilitate NET formation

Macrophages play a crucial role in orchestrating NET formation, particularly through pyroptosis-driven inflammatory signaling. Notably, NET accumulation correlates with macrophage pyroptosis, and elevated NET and caspase-1 levels are associated with the severity of ARDS [128]. Additionally, NETs promote macrophage pyroptosis in sepsis and chronic hepatic inflammation/fibrosis, further contributing to tissue damage [129, 130]. Conversely, pyroptotic macrophages generate microvesicles that stimulate NET formation both in vitro and in vivo through mitochondrial transfer, establishing a feedback loop that amplifies inflammation [131] (Fig. 4).

Beyond pyroptosis, macrophages also drive NET formation through cytokine secretion. Exosomes enriched in miR-146a, derived from oxidized low-density lipoprotein (oxLDL)-treated macrophages, suppress superoxide dismutase 2 (SOD2), leading to increased ROS production and NET release, accompanied by pro-inflammatory cytokine secretion [132]. Additionally, macrophage nucleotide-binding oligomerization domain-like receptor protein 3 (NLRP3) inflammasomes, regulated by Bruton’s tyrosine kinase (BTK), promote NET formation, while spleen tyrosine kinase (SYK) signaling within macrophages can further enhance NET production via NLRP3 activation [133, 134].

The biological impact of macrophage-driven NET formation extends beyond inflammation. In co-culture systems with THP-1 and A549 cells, NETs enhance cancer cell invasion and migration, with their pro-metastatic effects partially dependent on cytokines secreted by macrophages, including IL-1β, IL-6, IL-18, and TNF-α [135]. These findings emphasize the macrophage-NET axis as a driver of both inflammatory and tumor-promoting processes.

#### NETs modulate macrophage polarization

NETs not only influence macrophage activation but also regulate macrophage polarization, shaping immune responses in various pathological settings. In severe and critical COVID-19 cases, increased NET concentrations drive macrophage activation, promoting the release of pro-inflammatory cytokines and exacerbating disease severity [136].

In the context of metabolic and cardiovascular diseases, NETs skew macrophages toward the M1 pro-inflammatory phenotype. In a diabetic mouse model of atherosclerosis, NETs enhance M1 polarization, fueling disease progression and highlighting NETs as a potential therapeutic target [137]. Similarly, in ventilator-induced lung inflammation, NETs facilitate M1 polarization by targeting hexokinase-2 (HK-2), further amplifying pulmonary inflammation [138].

However, NET-driven macrophage polarization remains reversible. Inhibition of PAD2 suppresses NET formation, promoting M2 macrophage polarization and thereby facilitating anti-inflammatory responses and tissue repair [139]. Conversely, M2 macrophages actively enhance NET clearance, further reinforcing their role in resolving inflammation [140]. Targeting key regulators such as AMPK, PAD2, and TLR9 offers a promising strategy to disrupt the pathological feedback loops between NET formation and macrophage polarization, with the potential to mitigate autoimmune diseases, inflammatory disorders, and cancer progression while preserving essential immune homeostasis.

## Targeting NETs for therapeutic intervention

With growing insights into the mechanisms governing NET formation, it has become increasingly evident that NETs play a pivotal role in the pathogenesis of inflammation-driven diseases, including autoimmune disorders, cancer progression, and thrombotic complications. Consequently, multiple therapeutic strategies have been developed to mitigate the adverse effects of NETs, focusing on three primary approaches: (i) inhibiting NET formation, (ii) promoting structural degradation, and (iii) regulating NET release (Table 2). These interventions aim to modulate aberrant NET activity while preserving their physiological roles in host defense.

**Table 2:** Inhibitors targeting NET inducers

Targets	Intervention	Diseases	Results	References
Targeting NET formation				
PAD4	Cl-amidine	Rheumatoid arthritis	Reduce inflammatory response and joint oedema	Schneider et al. [141]
		Inflammatory bowel disease	Relief of tissue inflammation	Zhang et al. [142]
		Type 1 diabetes	Reduce autoantibody production	Shen et al. [143]
		Breast cancer	Reduce number of lung metastases	Inoue et al. [144]
	BB-Cl-amidine	Systemic Lupus Erythematosus	Reduce proteinuria and immune complex	Knight et al. [173]
		Colon cancer	Reduce the number and size of liver metastases	Yuzhalin et al. [174]
	GSK484	Breast cancer	Reduce lung metastasis	Albrengues et al. [145]
		Ovarian cancer	Reduce cancer cell implantation in the omentum	Lee et al. [146]
	BMS-P5	Multiple myeloma	Prolongation of survival	Li et al. [147]
NE	Sivelestat	Atherosclerosis	Reduce lipid deposition and inflammation in the aorta	Nie et al. [148]
		Inflammatory bowel disease	Reduce colitis activity index	Shioya et al. [149]
		Psoriasis	Reduce T-lymphocyte infiltration in the skin	Zhukov et al.[150]
		Colon cancer	Reduce tumor colonization in the lungs and liver	Rayes et al. [151]
	GW311616A	Lung cancer	Reduce tumor cell metastasis in the liver	Rayes et al. [151]
		Diffuse large B cell lymphoma	Retard tumor progression	Nie et al. [35]
	CHF6333	Bronchiectasis	Reduce lung tissue infection and inflammation	Gramegna et al. [152]
MPO	PF-1355	Glomerular basement membrane disease	Suppression of chronic renal insufficiency	Zheng et al. [153]
		Myocarditis	Improve cardiac function and reduce inflammatory infiltration	Chen et al. [155]
	INV-315	Atherosclerosis	Reduce plaque load and improve endothelial function	Shamsuzzaman et al. [154]
	AZM198	Crescentic glomerulonephritis	Reduce endothelial cell injury	Antonelou et al. [175]
	NBD peptide	Breast cancer	Inhibit tumor growth and progression	Zhu et al. [176]
CXCR1/2	Reparixin	Breast cancer	Enhancement of anti-tumour effect in combination with immune checkpoint inhibitors	Teijeira et al. [48]
	AZD5069	COPD and asthma patients	Reduce airway inflammation	Galoș et al. [157]
Autophagy	Chloroquine	Cancer-related thrombosis	Reduce the incidence of venous thromboembolism in the peri-operative period	Boone et al. [156]
	Wortmannin	Acute promyelocytic leukemia	Inhibit LC3 aggregation and ET release	Ma et al. [82]
Targeting NET depletion				
DNA	DNase I	Colitis	Attenuate colitis as well as colitis-associated tumorigenesis	Li et al. [160]
		Rheumatoid arthritis	Reduce endothelial dysfunction and immune cell activation	Pérez-Sánchez et al. [161]
		Lung cancer	Reduce adhesion of tumor cells to the hepatic sinusoids	Najmeh et al. [159]
		Hepatocellular carcinoma	Reduce hepatocellular carcinoma metastasis	Yang et al. [164]
		Breast cancer	Reduced lung metastases	Park et al. [162]
		Breast cancer	Prevention of thrombosis	Várady et al. [165]
		Pancreatic ductal adenocarcinoma	Reduce the number and size of liver metastatic nodules	Takesue et al. [163]
Targeting NET release				
ROS	NAC	Biliary atresia	Mice exhibited delayed disease onset and reduced morbidity and mortality	Zhang et al. [166]
	DPI	Septic	Reduce cytotoxicity	Kumar et al. [167]
	DNP	Thrombocytopenia	Reduce thrombosis in thrombocytopenic patients	Leung et al. [168]
	AZD7986	Breast cancer	Reduction of lung metastases	Xiao et al. [169]
CitH3	rhThrombomodulin	Sepsis	Inhibition of endotoxin-induced acute kidney injury	Harada et al. [170]
		Septicaemia	Limit immunothrombotic response and reduce lung damage	Helms et al. [171]
		Pancreatic cancer	Prevent pancreatic cancer metastasis to the liver	Kajioka et al. [172]
	Zinc	Zinc deficiency	Maintain efficient functioning of the innate immune response	Kuźmicka et al. [177]

### Inhibiting NET formation

#### PAD4 inhibition

PAD4-mediated histone citrullination is a key driver of chromatin decondensation during NET formation. Pharmacological inhibition of PAD4, using agents such as Cl-amidine and GSK484, has been shown to markedly attenuate inflammatory responses, slow autoimmune disease progression, and suppress cancer metastasis across a range of preclinical models (Table 2). Notably, BMS-P5 exhibits superior PAD4 inhibitory potency and selectivity, with favorable oral bioavailability and minimal off-target effects, making it a promising candidate for clinical application [173]. Although these inhibitors demonstrate broad therapeutic potential, significant challenges remain in achieving isoform specificity and minimizing immune dysregulation, underscoring the need for further structural optimization to enable successful clinical translation.

#### NE inhibition

NE facilitates chromatin decondensation and nuclear membrane rupture during NET formation. Clinically approved agents such as Sivelestat have been shown to alleviate NET-driven pathologies, including acute respiratory distress syndrome (ARDS) and sepsis-related organ injury. Emerging inhibitors, such as GW311616A and CHF6333, demonstrate improved pharmacokinetic profiles and anti-tumor efficacy in preclinical models of lung cancer and lymphoma. However, issues related to oxidative stability and long-term safety remain to be fully addressed (Table 2).

Despite their promise, several challenges persist in optimizing NE inhibitors for clinical use. These include stability and efficacy under oxidative stress conditions, the need for high specificity to minimize off-target effects, and the requirement for long-term safety evaluations.

#### MPO inhibition

MPO generates oxidants essential for NET formation. Irreversible inhibitors, such as AZM198, achieve near-complete MPO inhibition with minimal off-target effects, while reversible agents like INV-315 reduce atherosclerotic plaque burden by modulating endothelial function. However, the clinical utility of these therapies remains constrained by systemic toxicities, such as PF-1355–induced tachycardia, and an incomplete mechanistic understanding of Compound II–enhancing agents (Table 2).

#### CXCR1 and CXCR2 inhibition

CXCR1/2 antagonists, such as Reparixin and SX-682, inhibit neutrophil recruitment to TMEs and sites of inflammation. Preclinical studies have demonstrated their dual benefits: enhancing anti-tumor immunity through the suppression of myeloid-derived suppressor cells (MDSCs) and reducing chronic inflammation. Ongoing clinical trials are investigating their potential synergy with immune checkpoint inhibitors in the treatment of metastatic cancers (Table 3).

**Table 3:** Clinical trials investigating NET-targeted therapeutic strategies

Medicines	Diseases	Clinical Trial	Status	Clinical significance
Sivelestat	ARDS	NCT06387823	Recruiting	Efficacy and safety of Sivelestat sodium and Dexamethasone in the treatment of ARDS
	ARDS	NCT04973670	Unknown	Protective effect of Sivelestat sodium on ARDS in patients with Sepsis
	Lung injury after esophagectomy	NCT01170845	Completed	The incidence of postoperative acute lung injury was significantly lower
	Acute lung injury	NCT00219375	Completed	Efficacy and safety of Sivelestat sodium hydrate in acute lung injury associated with systemic inflammatory response syndrome
	Acute lung injury	NCT00036062	Completed	To determine the efficacy and safety of Sivelestat in subjects with acute lung injury
	Acute Aortic Dissection	NCT05874700	Not yet recruiting	A pilot study of Sivelestat sodium to shorten mechanical ventilation in acute aortic dissection
	Pulmonary dysfunction and organ dysfunction after cardiovascular surgery	NCT06195267	Recruiting	Evaluating the protective effects of Sivelestat against pulmonary dysfunction and organ dysfunction after cardiovascular surgery
SX-682	Myelodysplastic syndrome	NCT04245397	Recruiting	To evaluate the efficacy and safety of SX-682 in treating MDS patients
	Multiple myeloma	NCT06622005	Recruiting	To evaluate the safety of SX-682 in combination with standard of care for the treatment of Carfilzomib, Daratumumab-Hyaluronidase and Dexamethasone for the safety of patients with relapsed refractory MM
	Metastatic melanoma	NCT03161431	Recruiting	To determine the safety profile of SX-682 alone and in combination with pembrolizumab in subjects with metastatic melanoma
	Pancreas cancer	NCT05604560	Recruiting	Evaluating the safety and clinical activity of tislelizumab in combination with SX-682 in subjects with newly diagnosed and surgically resectable pancreatic cancer
	Pancreatic ductal adenocarcinoma	NCT04477343	Recruiting	To evaluate the safety and tolerability of SX-682 in combination with tolerability as maintenance therapy in patients with metastatic ductal adenocarcinoma of the pancreas
	Advanced solid tumors	NCT04574583	Completed	6 out of 10 patients were maintained in disease stabilization
	Non-small cell lung cancer	NCT05570825	Recruiting	To evaluate the efficacy and safety of SX-682 versus Pembrolizumab in patients with metastatic or recurrent stage IIIc or stage IV non-small-cell lung cancer
	Prostate cancer	NCT06228053	Recruiting	To evaluate the efficacy of SX-682 in combination with Enzalutamide in the treatment of men with abiraterone-resistant metastatic calcification-resistant prostate cancer
Navarixin	Non-small cell lung cancer, Prostate cancer, Colorectal cancer	NCT03473925	Completed	Of 105 patients, 3 had a partial response. Median progression-free survival was 1.8–2.4 months
	Asthma	NCT00688467	Completed	No significant difference in efficacy compared to placebo in 13 patients
	Neutrophilic asthma	NCT00632502	Completed	A mean reduction of 36.3% in sputum neutrophil percentage
	Psoriasis	NCT00684593	Completed	Only 8 out of 21 patients showed slight improvement in symptoms
AZD5069	Uncontrolled persistent asthma	NCT01704495	Completed	No reduction in the frequency of severe exacerbations in patients with uncontrolled severe asthma
	Chronic Obstructive Pulmonary Disease	NCT01233232	Completed	58 patients were generally well tolerated, with no increase in the incidence of infections
	Bronchiectasis	NCT01255592	Completed	19 patients had significantly lower neutrophil counts
	Metastatic castration resistant prostate cancer	NCT03177187	Terminated	To find out the side effects and safety of a combination of the CXCR2 antagonist, AZD5069 in combination with the androgen receptor antagonist, enzalutamide in patients with metastatic castration resistant prostate cancer
Pulmozume	Ischaemic stroke	NCT05203224	Recruiting	Improving early reperfusion with adjuvant dornase alfa in large vessel ischemic stroke
	Ischaemic stroke	NCT04785066	Recruiting	Efficacy of Pulmozyme on arterial recanalization in post-thrombectomy patients managed for ischemic stroke
	Respiratory distress syndrome	NCT03368092	Recruiting	Inhaled dornase alpha to reduce respiratory failure after severe trauma
	COVID-19 respiratory failure	NCT04445285	Recruiting	Using rhDNase to reduce mortality in COVID-19 patients with respiratory failure
	Pleura empyema	NCT04095676	Recruiting	VATS surgery compared to drainage in the treatment of pleural empyema
	Cystic fibrosis	NCT04468100	Completed	Supporting the clinical Tigerase biosimilarity to Pulmozyme administered in CF patients with severe impairment of pulmonary function
	Cystic fibrosis	NCT00265434	Completed	Dornase alfa improved quality of life
	Severe asthma	NCT00169962	Completed	rhDNAse did not cause clinical improvement among severely ill adults refractory to standardized care
	Neonatal ventilator-associated lung infections	NCT01356147	Completed	Oxygen requirement was reduced by an average of 16.5% from baseline in 6 patients
	Head and Neck Cancer	NCT00536952	Unknown	Evaluation of the efficacy of Pulmozyme in patients with head and neck cancer treated with radiotherapy and chemotherapy
Oshadi D	Acute myeloid leukemia or lymphoid leukemia	NCT02462265	Suspended	Evaluating the combination of Oshadi D and Oshadi R for remedial chemotherapy in patients with acute myeloid leukemia or lymphoid leukemia

#### Targeting autophagy to inhibit NET formation

Autophagy inhibitors, such as chloroquine, mitigate NET-driven thrombosis and inflammation in models of pancreatic cancer and vasculitis by suppressing LC3 aggregation and histone citrullination. However, their broad effects on cellular processes warrant caution, as balancing NET suppression with the preservation of essential autophagy functions remains a critical challenge.

### Promoting NET degradation

#### DNase I and its clinical applications

Exogenous DNase I degrades the DNA scaffolds of NETs, alleviating inflammation in models of rheumatoid arthritis and colitis. Although its efficacy in SLE is limited due to resistance from protein-bound NET complexes, DNase I has demonstrated anti-metastatic effects by disrupting tumor cell adhesion within hepatic sinusoids (Table 2). Clinical repurposing of Pulmozyme (recombinant human DNase I) shows promise in reducing cancer-associated thrombosis and metastasis (Table 3).

#### Enhancing endogenous DNase activity

A novel therapeutic strategy involves enhancing endogenous DNase function rather than relying solely on exogenous enzymatic degradation. A study by Ondracek et al. has revealed that regular physical activity enhances cardiovascular fitness while reducing circulating free DNA (cfDNA) and upregulating endogenous DNase activity, suggesting a non-pharmacological approach to NET clearance [178].

### Regulation of NET release

#### Targeting ROS to suppress NET formation

Antioxidants, such as N-acetylcysteine (NAC) and diphenyleneiodonium (DPI), along with cathepsin C (CTSC) inhibitors like AZD7986, reduce ROS-driven NET formation, improving outcomes in models of sepsis and cancer metastasis. Combinatorial approaches, such as exenatide combined with anti-PD-1 therapy, further enhance immunotherapy efficacy by suppressing NET-mediated immunosuppression (Table 2).

#### Histone citrullination

Thrombomodulin and zinc chelators inhibit citrullinated histone H3, thereby attenuating immunothrombosis in sepsis and pancreatic cancer. Interestingly, zinc supplementation paradoxically stabilizes NET dynamics, preserving innate immune function while reducing pathological NET release (Table 2).

## Discussion and conclusion

Despite considerable advancements in elucidating the molecular mechanisms governing NET formation, several aspects remain inadequately understood, particularly the precise activation of PAD4 and its cooperative role in NET release. Additionally, NETs exert profound regulatory effects on immune cell activation, differentiation, and function. Unraveling the intricate crosstalk between NETs and immune components across various pathological contexts represents a key direction for future research.

The regulatory and therapeutic implications of NETs are increasingly being recognized. However, achieving precise modulation of NET formation and function, and translating these insights into clinical interventions, remains a formidable challenge. Future research efforts should focus on developing tailored intervention strategies that align with the pathophysiological characteristics and disease progression stages. In early-stage infections, enhancing the antimicrobial efficacy of NETs to facilitate rapid pathogen clearance should be prioritized. Conversely, in conditions characterized by sterile inflammation or advanced malignancies, strategies should aim to curtail excessive NET formation to mitigate tissue damage and tumor progression.

A critical aspect of NET-targeted therapies should be the regulation of their formation rather than merely eliminating preformed NETs. This can be accomplished by identifying and selectively targeting key molecular mediators and signaling pathways involved in the early phases of NET generation, such as PAD4, NOX, and autophagy-associated proteins. Such approaches may pave the way for novel therapeutic strategies to control NET formation effectively.

Furthermore, the interplay between NETs and other immune system components warrants further investigation, particularly concerning its impact on immune response quality and efficacy. A deeper understanding of NET’s role in immune regulation could provide valuable insights for advancing precision medicine and developing targeted therapeutic strategies. These endeavors hold significant promise for enhancing treatment outcomes across a broad spectrum of diseases while driving scientific progress in immunology and related disciplines.

## Data Availability

No datasets were generated or analysed during the current study.

## Abbreviations

NETs: Neutrophil extracellular traps
TAN: Tumor-associated neutrophils
IFN: Interferon
TGF: Transforming growth factor
COVID-19: Corona Virus Disease 2019
NOX: Nicotinamide Adenine Dinucleotide Phosphate Oxidase
PMA: Phorbol 12-myristate 13-acetate
PKC: Protein kinase C
Raf-MEK-ERK-MAP: Rapidly Accelerated Fibrosarcoma-Mitogen-Activated Protein Kinase-Extracellular Signal-Regulated Kinase-Mitogen-Activated Protein Kinase
ROS: Reactive oxygen species
NE: Neutrophil elastase
MPO: Myeloperoxidase
PAD4: Peptidylarginine deiminase 4
CDK4/6: Cyclin-dependent kinase 4/6
RIPK3: Receptor-interacting serine/threonine-protein kinase 3
MLKL: Mixed lineage kinase domain-like
GSDMD: Gasdermin D
GM-CSF: Granulocyte–macrophage colony-stimulating factor
LPS: Lipopolysaccharide
C5a: Complement component 5a
ATP: Adenosine triphosphate
cGAS/STING: Cyclic GMP-AMP synthase/Stimulator of interferon genes
SLE: Systemic lupus erythematosus
3-MA: 3-Methyladenine
mTOR: Mechanistic target of rapamycin
PI3K: Phosphoinositide 3-kinase
ANCA: Anti-neutrophil cytoplasmic antibodies
UC: Ulcerative colitis
REDD1: Regulated in development and DNA damage responses 1
IL: Interleukin
TLR: Toll-like receptor
E. coli: Escherichia coli
S. aureus: Staphylococcus aureus
MAC-1: Macrophage-1 antigen
GPIbα: Glycoprotein Ib alpha
PD1: Programmed cell death protein 1
Tim3: T-cell immunoglobulin and mucin-domain containing-3
Lag3: Lymphocyte-activation gene 3
TNF-α: Tumor necrosis factor-alpha
Arg1: Arginase 1
TMCO6: Transmembrane and coiled-coil domains 6
NASH: Nonalcoholic steatohepatitis
OXPHOS: Oxidative phosphorylation
FOXP3: Forkhead box protein P3
Th: T helper
STAT6: Signal transducer and activator of transcription 6
BAFF: B-cell activating factor
ACPA: Anti-citrullinated protein antibodies
SKAP1: Src kinase-associated phosphoprotein 1
NFATc1/CXCL8: Nuclear factor of activated T cells 1/C-X-C motif chemokine ligand 8
DCs: Dendritic cells
COPD: Chronic obstructive pulmonary disease
NPMc +: Mutant nucleophosmin 1
MMP12: Matrix metalloproteinase-12
AMPK: Adenosine 5 ‘-monophosphate-activated protein kinase
ARDS: Acute respiratory distress syndrome
oxLDL: Oxidized low-density lipoprotein
SOD2: Superoxide dismutase 2
NLRP3: Nucleotide-binding oligomerization domain-like receptor protein 3
SYK: Spleen tyrosine kinase
BTK: Bruton's tyrosine kinase
HK-2: Hexokinase-2
HER2: Human epidermal growth factor receptor 2
DLBCL: Diffuse large B-cell lymphoma
AATD-LD: Alpha-1 antitrypsin deficiency-related liver disease
4-amino-TEMPO: 4-Amino-2,2,6,6-tetramethylpiperidine-1-oxyl
HClO: Hypochlorous acid
IC50: Half maximal inhibitory concentration
MDSC: Myeloid-derived suppressor cell
PDAC: Pancreatic ductal adenocarcinoma
CF: Cystic fibrosis
NAC: N-Acetyl-L-cysteine
DPI: Diphenylene Iodonium
DNP: 2,4-Dinitrophenol
CTSC: Cathepsin C
TSP-1: Thrombospondin-1
